# Does athletic identity enhance subjective wellbeing among elite collegiate athletes in team sports? A sequential mediation model of perceived team support and self-efficacy

**DOI:** 10.3389/fpsyg.2026.1816669

**Published:** 2026-05-26

**Authors:** Junhe Cui, Mingliang Zhang, Yankun Han, Weiqi Chai

**Affiliations:** 1 Department of Sport & Leisure Studies, Hoseo University, Asan-si, Republic of Korea; 2 College of Physical Education, Jilin Normal University, Siping, Jilin, China; 3 College of Physical Education, Beihua University, Jilin, Jilin, China; 4 Physical Education College, Jilin University, Changchun, Jilin, China

**Keywords:** athletic identity, perceived team support, self-efficacy, sequential mediation model, subjective wellbeing

## Abstract

To investigate the mediating roles of perceived team support and self-efficacy in the relationship between athletic identity and subjective wellbeing among elite collegiate athletes in team sports. A total of 309 valid questionnaires were collected from athletes across six universities in China. Structural equation modeling and bootstrap analysis were employed to test the sequential mediation model. Athletic identity exhibited a significant positive effect on subjective wellbeing. Perceived team support and self-efficacy independently mediated this relationship, while their sequential mediation effect was also significant. Perceived team support and self-efficacy sequentially mediated the positive association between athletic identity and subjective wellbeing. These findings highlight the importance of fostering supportive team environments and enhancing self-efficacy to promote mental health and holistic development in elite collegiate team-sport athletes.

## 1 Introduction

In recent years, discussions on mental health issues of elite college student-athletes have been increasing, and maintaining athletes' mental health has become a major challenge (Terry and Parsons-Smith, 2021). High-intensity physical confrontations, complex tactical strategies, enormous psychological pressure, and dynamic relationships with coaches for team sport athletes are potential triggers for athletes' psychological problems (KremŽar Jovanović et al., 2022). In addition, the psychological characteristics of elite athletes are associated with higher levels of emotion regulation, self-confidence, and self-concept (Durand-Bush and Salmela, 2002; Holt and Dunn, 2004; Oliver et al., 2010). Hoover et al. (2017) found in a study targeting basketball players that when experienced players express positive emotions, their shooting efficiency increases. Athletes'

emotional expression and emotion regulation abilities are key abilities for sports success (Sagar et al., 2009). Studies have pointed out that positive emotions can offset the impact of negative emotions, alleviate stress to achieve adaptation (Fredrickson and Branigan, 2005). Therefore, athletes should pay attention to both positive and negative emotions. Considering this concept, it is important to verify the role of athletes' subjective wellbeing, which includes life satisfaction, positive emotions, and negative emotions, and has been proven to be a key factor affecting sports performance and long-term satisfaction (Diener et al., 2018). Diener (2000) showed that people with higher wellbeing experience more positive emotions than negative emotions when engaging in activities they consider interesting and satisfying. Athletes with higher levels of subjective wellbeing exhibit stronger concentration, resilience, and tighter team cohesion (Peris-Delcampo et al., 2024). Therefore, among elite college student-athletes in team sports, understanding the level of athletes' subjective wellbeing is very important.

Meanwhile, there is a correlation between subjective wellbeing and athletic identity (Martin et al., 2014). Athletic identity is the degree to which an individual identifies with the athlete role and has been confirmed to play a key role in shaping athletes‘ self-perception, psychological experiences, and behavioral engagement (Lamont-Mills and Christensen, 2006). However, this strong identification with the athlete role may also generate negative emotions for athletes, especially for groups with strong identification with the athlete role, establishing and consolidating a multi-dimensional identity for self-identity may face challenges (Mathews et al., 2021). For example, strong identification with the athlete role may lead to the formation of a unidimensional identity focusing solely on athletic ability. When athletes primarily define themselves as the athlete role without exploring interests or roles beyond the athlete role, a unidimensional identity is formed (Verkooijen et al., 2012). Sport-centered unidimensional identity is associated with reduced levels of subjective wellbeing (Tyrance et al., 2013). Athletes with a unidimensional identity tend to rely on evaluations of athletic performance to determine self-worth, self-esteem, and confidence (Verkooijen et al., 2012). For such athletes, adverse athletic outcomes, such as poor athletic performance, injury, or withdrawal from sports, may lead to a decline in self-worth and subsequently trigger a reduction in subjective wellbeing (Brewer et al., 1993). In elite college team sports, this duality presents significant challenges to athletes' growth and development. Therefore, understanding how athletic identity in elite college team sports affects subjective wellbeing, under what conditions it promotes or hinders athletes‘ wellbeing, is of great significance for promoting athletes' comprehensive development.

The relationship between athletic identity and subjective wellbeing is complex. Although some studies have pointed out a positive correlation between the two, that is, stronger athletic identity can bring a sense of purpose and belonging (Watson et al., 2024), many studies have also revealed its potential negative factors, where this identity may trigger anxiety, depression, and even reduce life wellbeing when athletic performance is threatened or identity is challenged (Senecal and Williams, 2024). Based on the above studies, the direct association between athletic identity and subjective wellbeing is likely mediated by other social factors. In addition, existing literature mostly focuses on single sports, lacking targeted research on the competitive pressure of elite college team sport athletes. There is still a significant gap in research in this area, and this duality highlights the necessity of studying mediating variables.

To address this gap, this study introduces “perceived team support” as a key mediating variable. Drawing on Social Support Theory (Cohen and Wills, 1985), perceived organizational support (Eisenberger et al., 1986), and Social Identity Theory (Turner et al., 1979), we define perceived team support as an athlete's subjective assessment of the value, respect, and backing received from teammates and coaches. Conceptualized as a vital social resource, robust team support significantly enhances wellbeing and belongingness. Social Identity Theory posits that individuals are motivated to maintain a positive self-concept; thus, favorable evaluations of one's team foster self-esteem (Turner et al., 1979). Furthermore, social comparisons reinforce group identification, further bolstering self-worth. Within team sports, perceived support from coaches and peers mitigates stress, cultivates positive self-concepts, and reinforces the adaptive dimensions of athletic identity (Russell, 2021). By shifting the analytical focus from isolated individual identity to dynamic socio-environmental interactions, this mediation model offers a more holistic explanation for the mechanisms underlying elite collegiate athletes' wellbeing.

Besides social factors, self-efficacy is an individual's confidence in the ability to take actions needed to achieve specific goals, which is currently widely recognized as a key psychological mechanism influencing wellbeing (Koçak, 2020). In the field of sport psychology, self-efficacy is closely related to motivation, performance persistence, and emotion regulation (Feltz et al., 2008). Athletes with a strong sense of athletic identity naturally possess the motivation for excellent performance, but this motivation can only be transformed into positive wellbeing when athletes also have high self-efficacy—that is, believing in their ability to cope with athletic challenges, perform skills under pressure, and recover from setbacks (Lpez-Rodriguez et al., 2025). Conversely, athletes with a strong sense of athletic identity but weak self-efficacy may face long-term stress, anxiety, and a lack of achievement, severely damaging their athletic self-efficacy (Huang et al., 2016). Therefore, self-efficacy, as a key internal mechanism, determines whether a strong sense of athletic identity becomes a force for success or a source of pressure.

**Figure 1:**
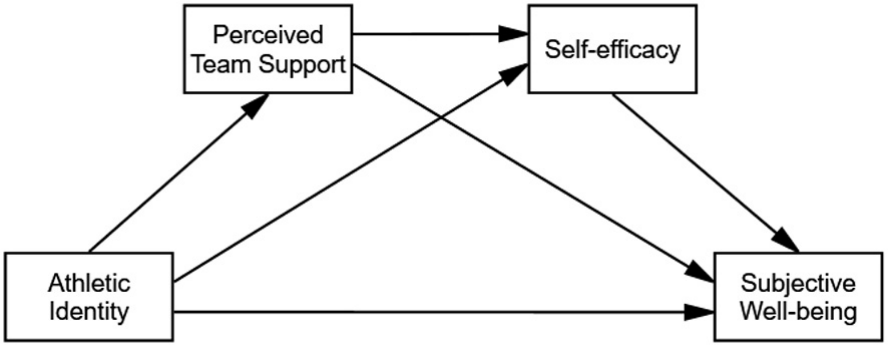
Proposed sequential mediation model.

In conclusion, the interaction between athletic identity and subjective wellbeing is not a direct link but is likely mediated by psychosocial mechanisms such as perceived team support and self-efficacy. Although existing studies have independently examined these constructs, none has integrated them into a mediating model specific to team sports. A “team” in sport science literature typically refers to a group of interdependent individuals who share a common goal, engage in coordinated interactions, and collectively represent an organizational unit. In the context of the present study, we operationally define a “team” as the cohort of elite collegiate athletes competing in the same sport discipline (e.g., basketball, volleyball) within the same university. While, understanding these pathways is crucial for developing measures to boost the competitive performance of elite college student-athletes in team sports and improve their mental health. Drawing on Diener et al. (1985) conceptualization of subjective wellbeing and aligning with the focus of our study, we have operationalized life satisfaction as a key component of subjective wellbeing. Figure 1 shows our proposed sequential mediation model. Meanwhile, we proposed four research hypotheses, namely athletic identity exerts a significant positive effect on subjective wellbeing among elite collegiate team-sport athletes; perceived team support mediates the relationship between athletic identity and subjective wellbeing; athlete self-efficacy mediates the relationship between athletic identity and subjective wellbeing; perceived team support and athlete self-efficacy sequentially mediate the relationship between athletic identity and subjective wellbeing.

From a practical standpoint, this study holds significant guiding value for coaches of team sports and program managers, thereby aiding the development of targeted intervention programs. By fostering a supportive team climate, such as implementing structured mentorship programs, establishing regular team-building workshops and implementing psychological skill training, such as cognitive restructuring via cognitive behavioral therapy, and mindfulness-based stress reduction aimed at enhancing self-efficacy, relevant practitioners can help elite college student-athletes in team sports develop a healthy and sustainable athletic identity. Such an identity not only fosters their long-term subjective wellbeing and career development but also mitigates potential negative impacts.

## 2 Literature review

### 2.1 Athletic identity and subjective wellbeing

Sports psychology began to focus on athletic identity in the 1990s (Lochbaum et al., 2022). Brewer et al. (1993) defined it as the degree to which athletes identify with their athletic role, which is closely related to the evaluation of self-definition, self-worth, and self-esteem. Caravaglia et al. (2023) argued that athletic identity is a mode of self-cognition or a belief system, which may have positive or negative effects on athletes' physical and mental health. Furthermore, Maike et al. (2025) posited that athletic identity is not only the degree of cognitive acceptance, intensity of emotional investment, and consistency of behavioral practice of an individual toward the athlete role, but also a unique self-cognitive label formed by an individual in the athletic context. Identity includes an individual's subjective cognition of identity and the positioning of relationships with others in the social environment (Brewer and Gardner, 1996; Caza et al., 2018). Research has shown that whether athletes or non-athletes, having a strong sense of athletic identity is related to physiological and psychosocial dimensions (Fraser, 2012).

Subjective wellbeing is comprising affective responses, satisfaction, and life satisfaction, reflecting an individual's assessment of current happiness (Silva et al., 2020). Subjective wellbeing includes personal life satisfaction, the predominance of desirable emotions, and the absence of undesirable emotional traits (Diener, 1984; Diener et al., 2018, 2003). Subjective wellbeing is an important evaluation indicator of athletic identity, and its importance is increasingly recognized by academia (Mooney et al., 2024). Most research results indicate that strong identification with the athlete role has a positive impact on sports achievement (Lamont-Mills and Christensen, 2006), self-confidence (Ryska, 2002), and social connection (Chen et al., 2010). For example, Uroh and Adewunmi (2021) argued that student-athletes who maintain a high sense of athletic identity exhibit fewer depressive symptoms than those who do not, because it can endow them with a sense of purpose, a sense of organization, and a sense of connection with sports (Edison et al., 2021). Graupensperger et al. (2020a) found that people with a strong sense of athletic identity who actively participate in high-intensity sports such as competitive sports tend to have better mental health, and this phenomenon may be an improvement in subjective satisfaction. Ryff (2013) emphasized that this identity is based not only on personal subjective feelings but also influenced by teammates, coaches, parents, and fans. Furthermore, a strong athletic identity also has positive significance for student-athletes during the transition period after retirement, as they can utilize traits such as resilience and competitiveness to stand out in their careers and increase wellbeing (Kidd et al., 2018; Menke and Germany, 2019). However, although most scholars have conducted positive discussions on athletic identity, they have overlooked that identity has duality and dynamics; therefore, some scholars believe that the view that athletic identity can generally enhance athletes' subjective wellbeing may have omissions. Empirical studies have highlighted this issue. For example, Grove et al. (1997) and Martin et al. (2014) argued that subjective wellbeing is negatively correlated with athletic identity. Strong identification with the athlete role may lead to the formation of a unidimensional identity, that is, focusing solely on athletic ability (Mathews et al., 2021). When athletes primarily define themselves as the athlete role and fail to explore interests beyond the athlete role, a unidimensional identity is formed (Verkooijen et al., 2012), and this cognitive pattern is associated with a decline in subjective wellbeing (Brewer et al., 1993; Tyrance et al., 2013). This identity often relies on athletic performance to predict self-worth and confidence, while negative outcomes such as poor athletic performance and injuries will lead to a decrease in self-worth, which in turn triggers a reduction in subjective wellbeing (Brewer et al., 1993; Martin et al., 2014; Tyrance et al., 2013; Verkooijen et al., 2012).

The above research findings indicate that the influence of athletic identity on subjective wellbeing is situationally dependent and constrained by specific conditions. In this context, whether the athletic identity of elite collegiate athletes in team sports impacts subjective wellbeing remains to be discussed, requiring further examination. To fill this gap, this study proposes the hypothesis that the athletic identity of elite collegiate athletes significantly affects subjective wellbeing in team sports.

### 2.2 The mediating role of perceived social support

Social support is defined as the assistance provided by people when helping others cope with life challenges and situational demands (Xu and Burleson, 2001). For college student-athletes, social support has a wide range of sources and may receive support from sports departments, coaches, team doctors, teammates, athletic trainers, friends, family, etc. (van Raalte and Posteher, 2019). These supports are to cope with athletes' physiological, psychological, and social pressures. Social support is a multidimensional concept, mainly including five forms, emotional support, esteem support, informational support, network support, and tangible support (Barrera Jr and Ainlay, 1983). For student-athletes, social support plays a positive role in various situations (Rees and Hardy, 2000). For example, when athletes are in low spirits due to poor sports performance, emotional support is most effective; while when athletes are in a slump, esteem support can play a key role (Rees and Hardy, 2000). In addition, the support from athletic trainers during athletes' injury periods (Yang et al., 2010), coaches' affirmative communication (Zourbanos et al., 2011), and recognition (Kristiansen and Roberts, 2010) all play a key role.

Research has shown that social support can significantly enhance individuals‘ life satisfaction and wellbeing, especially support from family, friends, and coaches (Mira et al., 2023). Social support is not only an important predictor of mental health (Ganster and Victor, 1988), but also plays a key role in ensuring individuals' healthy development (Viner et al., 2012). Social support also promotes positive emotions and avoids anxiety by influencing emotions, cognitions, and behaviors (Gallagher and Vella-Brodrick, 2008), thereby becoming a necessary prerequisite for subjective wellbeing and thus influencing individuals‘ subjective wellbeing (Guo et al., 2024). In addition, high levels of social support can enhance individuals' sense of being understood and respected, help maintain emotional stability, and ultimately enhance subjective wellbeing (Fredrickson, 2001). Throughout the careers of college student-athletes, social support is regarded as an indispensable element for their sports success. Although most studies have shown that social support has a positive impact on student-athletes, social support may depend on specific situations, the type of support delivered, and the identity support system of the provider (van Raalte and Posteher, 2019). Based on the above discussions, this study proposes the following hypothesis: perceived social support of elite collegiate athletes mediates the relationship between identity and subjective wellbeing in team sports.

### 2.3 The mediating role of self-efficacy

Self-efficacy is an individual's confidence and belief in completing specific tasks or behaviors (Bandura, 1994). Self-efficacy determines an individual's activity choices, persistence in overcoming difficulties, effort level, and performance level (Bandura, 1994). Individuals with higher self-efficacy in difficult situations will exert more effort to achieve goals, not easily give up when facing obstacles, and persevere to the end (Kiremit and Gokler, 2010). Sport self-efficacy refers to an individual's belief in their own athletic ability, reflecting the athlete's confidence level in using skills to achieve specific sports goals, and the subjective assessment of an individual's ability to control their own sports behavior and performance (Coopersmith, 1965). When student-athletes encounter stressful events and feel anxious, their sense of competence may affect their academic and sports performance (van Raalte and Posteher, 2019). Athletes with high self-efficacy often exhibit stronger intrinsic motivation and self-confidence, enabling them to adopt positive strategies to cope with adversity and challenges, thereby demonstrating higher psychological resilience (Zhou et al., 2025). Research has shown that higher self-efficacy is positively correlated with wellbeing (Guo et al., 2024). A recent meta-analysis further corroborates this perspective (Cui et al., 2025). People with high self-efficacy can both properly handle positive and negative emotions and maintain optimistic expectations for the future, thereby comprehensively enhancing overall wellbeing (Tong, 2004). In addition, the stronger the athlete's self-efficacy, the higher the self-confidence, and the stronger the sense of control over the environment, the more significantly subjective wellbeing will be enhanced (Zheng and Yu, 2017). Conversely, people lacking sufficient self-efficacy may face the dilemma of vague goals or insufficient motivation, leading to a decline in subjective wellbeing (Zhang and Chen, 2025). Therefore, this study proposes the following hypothesis: self-efficacy of elite collegiate athletes mediates the relationship between identity and subjective wellbeing in team sports.

### 2.4 The chain mediation role of social support and self-efficacy

Research has shown that social support is positively correlated with self-efficacy (Zhang B. et al., 2023). When individuals perceive support and affirmation from others, they are more likely to believe in their ability to overcome difficulties and achieve goals, thereby strengthening self-efficacy (Lent et al., 1986). In addition, coaches‘ behaviors can significantly affect athletes' self-efficacy (Saville and Bray, 2016); when coaches continuously provide supportive feedback, athletes are more likely to perceive training experiences as successful and competence-enhancing processes, thereby enhancing perceived efficacy and wellbeing (Li et al., 2025). Individual factors such as self-efficacy also interact with environmental factors such as social support (Hu and Zhao, 2023). Specifically, individuals with higher self-efficacy are usually more confident and more inclined to proactively seek and effectively utilize existing social support resources to solve problems and boost confidence (Y. Zhang Y. et al., 2023). Meanwhile, social support itself is also an important environmental factor shaping self-efficacy (Rippon et al., 2024). Theoretically, when individuals obtain or perceive social support from classmates, colleagues, or teams in learning and work environments, this support becomes an important source for satisfying their need for belonging and helps them establish a strong sense of identity. Therefore, this study proposes the following hypothesis: social support and self-efficacy of elite collegiate athletes play a chain-mediating role in the relationship between identity and subjective wellbeing in team sports.

## 3 Methods

### 3.1 Procedure and participants

This study was conducted in Jilin Province, China. The researchers previously confirmed the number and names of universities with elite college student-athletes within the province, and contacted the persons in charge of sports competitions at the relevant institutions one by one to inform them in detail of the purpose, process, and related content of the study to obtain approval from the heads of the participating institutions. Ultimately, six institutions within the province agreed to participate in the experimental data collection of this study. Data collection was carried out in the form of offline paper questionnaires, taking 1 week. During this period, the researchers personally took the paper questionnaires to each institution for investigation one by one, assisted by two student assistants who helped distribute the questionnaires and the pencils used for filling them out, and the filling venues were all classrooms after the athletes' theoretical learning sessions. At the site, the researchers explained the purpose of the study and the use of the collected data to the athletes to ensure written consent from the participants. Meanwhile, the researchers explained on-site any questions or confusions arising during the questionnaire filling for the respondents. The average time for filling out the questionnaire was about 20 min. After completion, the student assistants collected, sorted, and archived the questionnaires on-site. Finally, a total of 322 questionnaires were distributed, and 322 were returned, of which 309 were valid, with an effective rate of 95.96%. This study was approved by the Academic Ethics Committee of the university. The demographic characteristics of the respondents are as shown in Table 1.

**Table 1:** Demographic characteristics.

Demographic	Number	Percent
Gender		
Male	223	72.17
Female	86	27.83
Age		
≤ 20	206	66.67
21–25	92	29.77
26–30	11	3.56
Years		
1–5	131	42.39
5–10	114	36.89
>10	64	20.71
Type		
Basketball	136	44.01
Football	109	35.28
Volleyball	61	19.74
Hockey	3	0.01

### 3.2 Measurements

We utilized scales that had been validated in prior research. As these scales were originally developed and reported in English-language academic papers, translation of their original English versions was necessary to develop a questionnaire that was both accurate and of high quality. To this end, we applied the back-translation method to create a Chinese adaptation of the instrument (Brislin, 1986).

### 3.3 Athletic identity

Adopted the 21-item Athletic Identity Questionnaire (Anderson, 2004), which is a first-order model containing four related factors: athletic appearance; importance of sports; competence; and encouragement. A 5-point Likert scale (1 = strongly disagree, 5 = strongly agree) was adopted. The original questionnaire showed good model fit, including NNFI = 0.96, CFI = 0.97, RMSEA = 0.058. The coefficient alpha reliabilities ranged from.78 to.89. In this study, this questionnaire was used to measure the extent to which elite college student-athletes identify with their athletic identity performance in training and competition, including athlete image; importance of sports; athlete competence; and encouragement received. Representative items include, for example, “I think I look athletic, like a person who exercises.” The Kaiser–Meyer Olkin (KMO) value was 0.968, with the Bartlett test of sphericity achieving statistical significance (*p* < 0.001).

### 3.4 Subjective wellbeing

Adopted a 5-item scale, namely the Satisfaction With Life (Diener et al., 1985), to measure the subjective wellbeing of elite college student-athletes. This scale is a 7-point Likert scale (1 = strongly disagree, 7 = strongly agree). The original questionnaire has good reliability, with a reliability coefficient of 0.87, and a two-month test-retest reliability coefficient of 0.82. The factor loadings of the items range from 0.61 to 0.84. The item-to-total correlation coefficients range from 0.57 to 0.75. In this study, this questionnaire was used to measure and ask about the extent to which elite college student-athletes feel their lives are fully satisfied compared with the standards they set for themselves. Representative items include, for example, “In most ways my life is close to my ideal.” The Kaiser–Meyer Olkin (KMO) value was 0.901, with the Bartlett test of sphericity achieving statistical significance (*p* < 0.001).

### 3.5 Perceived team support

Adopted an 8-item Perceived Team Support Scale (Sheng et al., 2010) to measure elite college student-athletes' perception of team support. This scale is a 7-point Likert scale (1 = strongly disagree, 7 = strongly agree). The original questionnaire's factor loadings range from 0.54 to 0.84, and in subsequent studies this questionnaire demonstrated good composite reliability, namely a CR value of 0.96, and a convergent validity AVE value of 0.73. In this study, this questionnaire was used to measure the extent to which elite college student-athletes perceive support from the team. Representative items include, for example, “My organization is willing to help me if I need a special favor.” The Kaiser–Meyer Olkin (KMO) value was 0.943, with the Bartlett test of sphericity achieving statistical significance (*p* < 0.001).

### 3.6 Athlete self-efficacy

Adopted the Athlete Self-Efficacy Scale (Koçak, 2020), which is a scale comprising 4 factors with a total of 16 items, the 4 factors being Sport discipline efficacy, Psychological efficacy, Professional thought efficacy, and Personality efficacy. Adopted a 5-point Likert scale (1 = strongly disagree, 5 = strongly agree). The original questionnaire showed good model fit, including NNFI = 0.96, CFI = 0.97, GFI = 0.91, RMSEA = 0.073. Cronbach's Alpha Reliability Coefficient was 0.898. In this study, this questionnaire was used to measure the extent to which elite college student-athletes believe in their ability to successfully complete various performance tasks related to sports. The model fit in the current study was also acceptable, with NNFI = 0.994, CFI = 0.997, GFI = 0.996, RMSEA = 0.082. Representative items include, for example, “I have the technical skills required for my sport discipline.” The Kaiser–Meyer Olkin (KMO) value was 0.970, with the Bartlett test of sphericity achieving statistical significance (*p* < 0.001).

### 3.7 Data analysis strategy

First, adopting the analytical strategy recommended by Fan et al. (2021), we used SPSS version 26.0 (SPSS Inc., USA) to calculate the Fleiss' Kappa values of each variable to assess interrater agreement. Calculated ICC (1) (intraclass correlation coefficient) to evaluate the intraclass correlations, and ICC (2) to evaluate the reliability of the group means (Bliese, 2000). If all data results meet the recommended reference values, it indicates that data aggregation is appropriate (Bliese, 2000). The athletic identity results indicated that ICC (1) is 0.709, ICC (2) is 0.981, and Fleiss's Kappa is 0.614. The subjective wellbeing results indicated that ICC (1) is 0.842, ICC (2) is 0.964, and Fleiss's Kappa is 0.759. The perceived team support results indicated that ICC (1) is 0.862, ICC (2) is 0.980, and Fleiss's Kappa is 0.801. The athlete self-efficacy results indicated that ICC (1) is 0.834, ICC (2) is 0.988, and Fleiss's Kappa is 0.812. Based on the above results, data aggregation can be performed; we aggregated individual-level data to the team level, namely team athletic identity, subjective wellbeing, perceived team support, and self-efficacy, to form team-level measures. Prior to formal data analysis, all data were standardized. The results showed that all Cronbach's alpha values ranged from 0.954 to 0.980 and exceeded 0.7, indicating satisfactory reliability (Tavakol and Dennick, 2011).

Next, we conducted descriptive statistical analysis on the data to present the demographic characteristics of the participants. Calculated Pearson's product-moment correlation to test the directions and correlations among all the variables. To verify the research hypothesis of this study, that perceived team support and athlete self-efficacy act as sequential mediating factors in the relationship between athletic identity and athlete subjective wellbeing, this study adopted the SPSS PROCESS macro, Model 6 (Preacher and Hayes, 2004), to test the stability and significance of the mediation effects. Particularly, we calculated 95% confidence intervals of the indirect effects derived from bias-corrected bootstrap estimates with 5,000 iterations, which are significant at *p* = 0.05 if the 95% confidence interval does not include zero.

There are two reasons for adopting this method. First, this method is widely applied in various research fields to verify the direct and indirect effects of mediation models. Second, this method is based on the non-parametric bootstrapping procedure. Non-parametric bootstrapping procedures are superior to traditional regression methods for testing indirect effects as the former do not make assumptions regarding the shape of the distribution of the variables or the sampling distribution (Preacher and Hayes, 2004). To calculate the indirect effects in this study, we selected Model 6 in PROCESS, which can be used to test the sequential multiple mediation model in this study, with athlete subjective wellbeing as Y, athletic identity as X, perceived team support as M1, athlete self-efficacy as M2, and 5,000 bootstrap resamples at bias-corrected with 95% confidence intervals.

To validate the hypothesized constructs, we estimated a measurement model using confirmatory factor analysis (CFA), where each observed indicator was specified to load on its corresponding latent construct, with correlations permitted among the constructs. Given the length of the Athletic Identity Questionnaire (21 items) and Athlete Self-Efficacy Scale (16 items), we employed item parceling to reduce model complexity and improve parameter estimation stability (Little et al., 2002). This approach aligns with recommendations for balancing model parsimony and construct representation (Matsunaga, 2008). Confirmatory factor analysis was conducted using IBM SPSS Amos 24. The model fit indices for the measurement model indicated an acceptable fit: χ^2^ = 425.013, df = 183, χ^2^/df = 2.322, RMSEA = 0.066, SRMR = 0.052, CFI = 0.976, TLI (NNFI) = 0.972, and GFI = 0.959.

Furthermore, we evaluated the composite reliability (CR) and construct validity. The composite reliability of the indicators should exceed the threshold value of 0.70 (Hair et al., 2009). Next, we computed the average variance extracted (AVE) to assess the convergent validity of the constructs. Theoretically, an AVE > 0.50 indicates that the variance captured by the construct is sufficient for the variables to converge into a single construct (Hair et al., 2009). Discriminant validity was evaluated by comparing the AVE to the squared correlation between latent constructs; discriminant validity is supported if the squared correlations between constructs are less than the AVE (Fornell and Larcker, 1981). The results presented in Tables 2, 3 indicated that the AVE of each construct exceeded 0.50, the composite reliability of the indicators exceeded 0.70, and the AVE of each construct was greater than the squared correlations between pairs of constructs, thereby supporting the construct validity.

**Table 2:** Confirmatory factor analysis results.

Construct	Item	Standardized factor loading	Cronbach' α	CR	AVE
AI	1.Appearance	0.848	0.954	0.949	0.824
	2.Importance	0.853			
	3.Competence	0.974			
	4.Encouragement	0.948			
ASE	1.Professional thought efficacy	0.910	0.972	0.973	0.900
	2.Personality efficacy	0.972			
	3.Sport discipline efficacy	0.973			
	4.Psychological efficacy	0.938			
PTS	1. My organization really cares about my wellbeing	0.942	0.980	0.983	0.880
	2. My organization strongly considers my goals and values	0.948			
	3. My organization shows little concern for me.	0.963			
	4. My organization cares about my opinions	0.951			
	5. My organization is willing to help me If I need a special favor.	0.942			
	6. Help is available from my organization when I have a problem	0.936			
	7. My organization would forgive an honest mistake on my part.	0.931			
	8. If given the opportunity, my organization would take advantage of me.	0.889			
SWB	1. In most ways my life is close to my ideal.	0.923	0.964	0.959	0.825
	2. The conditions of my life are excellent.	0.956			
	3. I am satisfied with my life	0.961			
	4. Sofar I have gotten the im-portant things I want in life	0.920			
	5. If I could live my life over, I would change almost nothing.	0.767			

AI-Athlete identity, ASE-Athlete self-efficacy, PTS– Perceived team support, SWB-Subjective wellbeing.

**Table 3:** Correlation matrix with the square root of AVE.

Variable	Mean	SD	AI	ASE	PTS	SWB
AI	4.030	0.788	0.908			
ASE	4.299	0.640	0.411^**^	0.949		
PTS	4.139	0.792	0.664^**^	0.406^**^	0.938	
SWB	4.164	0.761	0.717^**^	0.452^**^	0.776^**^	0.908

AI-Athlete identity, ASE-Athlete self-efficacy, PTS– Perceived team support, SWB-Subjective wellbeing, ^**^ *p* < 0.01.

## 4 Results

To test the hypothesis that perceived team support (PTS) and athlete self-efficacy (SEF) sequentially mediate the effect of athletic identity (AI) on athlete subjective wellbeing (SWB), we conducted a sequential mediation analysis (Model 6 in PROCESS) using bootstrap resampling methods. The results, presented in Table 4, indicated that the total effect of AI on SWB was significant (β = 0.692, *t* = 18.032, *p* < 0.001). The direct effect of AI on SWB excluding the mediating effects of PTS and SEF was also significant (β = 0.322, *t* = 7.596, *p* < 0.001); therefore, Hypothesis 1 was supported. Moreover, all direct effects of independent variables on the dependent variable in the model were positive and significant.

**Table 4:** Direct effects of independent variables on dependent variables.

IV	DV	Coefficient	SE	T	p	LLCI	ULCI
AI	PTS	0.667	0.043	15.550	< 0.001	0.582	0.751
	ASE	0.205	0.055	3.699	< 0.001	0.096	0.314
	SWB^*^	0.322	0.042	7.596	< 0.001	0.239	0.406
PTS	ASE	0.193	0.055	3.490	0.001	0.084	0.301
	SWB	0.491	0.042	11.663	< 0.001	0.408	0.574
ASE	SWB	0.128	0.043	2.985	0.003	0.043	0.212
Total effect of AI on SWB		0.692	0.038	18.032	< 0.001	0.617	0.768

AI-Athlete identity, ASE-Athlete self-efficacy, PTS- Perceived team support, SWB-Subjective wellbeing, ^*^-Direct effect.

Moreover, the total indirect effect was statistically significant [β= 0.37, *SE* = 0.057, 95% CI (0.269, 0.495)]. Specifically, the specific indirect effect via perceived team support (PTS) mediating the impact of athletic identity (AI) on athlete subjective wellbeing (SWB) was significant [β= 0.327, *SE*= 0.060, 95% CI (0.227, 0.463)]. The specific indirect effect via athlete self-efficacy (SEF) mediating the impact of AI on SWB was also significant [β= 0.026, *SE*= 0.015, 95% CI (0.002, 0.060)]. Therefore, Hypotheses 2 and 3 were supported (Table 5).

**Table 5:** Indirect effects of AI on SWB.

Indirect effect key:	Effect	Bootse	BootLLCI	BootULCI
AI → PTS → SWB(Ind1)	0.327	0.060	0.227	0.463
AI → ASE → SWB(Ind2)	0.026	0.015	0.002	0.060
AI → PTS → ASE → SWB(Ind3)	0.016	0.009	0.002	0.037
C1 Ind1 minus Ind2	0.301	0.068	0.183	0.451
C2 Ind1 minus Ind3	0.311	0.060	0.213	0.447
C3 Ind2 minus Ind3	0.010	0.015	−0.018	0.043

AI-Athletic identity, ASE-Athlete self-efficacy, PTS– Perceived team support, SWB-Subjective wellbeing.

**Figure 2:**
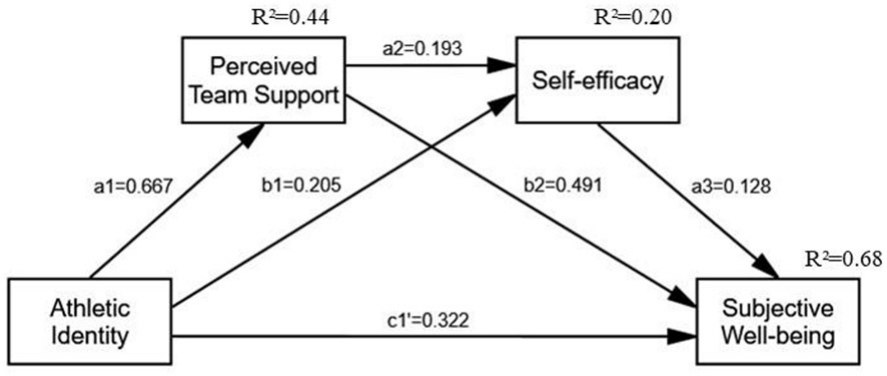
The results of the sequential model with path coefficients.

To examine the sequential multiple mediation effect, the results indicated that the specific indirect effect of athletic identity (AI) on athlete subjective wellbeing (SWB) through both perceived team support (PTS) and athlete self-efficacy (SEF) was significant, with a point estimate of 0.016 and a 95% confidence interval ranging from 0.002 to 0.037, providing full support for Hypothesis 4 (Table 5). The results indicated that PTS and SEF serve as sequential mediators in the relationship between AI and SWB. However, a comparison of specific and sequential mediation effects revealed, as shown in Table 5, that the specific indirect effect via PTS was the strongest. Although the sequential mediation effect existed, compared with the specific indirect effect via PTS, PTS played a better mediating role in the relationship between AI and SWB. The potential reasons for this and analysis of corresponding results are detailed in the subsequent discussion section. All results are visually presented in Figure 2.

## 5 Discussion

The purpose of this study was to explore the sequential mediating effect of perceived team support and self-efficacy of elite college student-athletes in team sports in the relationship between athletic identity and subjective wellbeing. The following section discusses the corresponding research findings.

First, through surveys of multiple team sports, this study confirmed that the athletic identity of athletes in team sports has a significant positive association with their subjective wellbeing. This enriches theoretically researchers' understanding of this pair of relational entities in team sports. Although previous studies have explored these two variables, few studies have treated these two variables as a pair of relational entities and conducted empirical analysis against the background of multiple team sports. Therefore, the results of this study make up for the deficiency of existing literature in the study of this pair of relational entities to a certain extent. Theoretically speaking, an athlete's identity recognition in team sports is the degree of recognition of the role they play, which determines their emotional investment in participating in and integrating into a sport. A strong sense of identity will enable athletes to feel the joy of active participation during their involvement in sports (Pavot and Diener, 2008). This is consistent with the results of this study. Furthermore, situating our findings within China's collectivist sporting culture reveals that athletic identity is deeply embedded in national prestige and team obligations rather than individual achievement alone. This cultural context likely amplifies the association between the variables examined.

However, in a survey of elite college soccer players, Mathews et al. (2021) found that there were differences in the impact of athletic identity on subjective wellbeing across athletic levels. For example, first-class athletes were more inclined to identify with their identity, but compared with third-class athletes, they had lower subjective wellbeing. This contradicts the results of this study. The possible reason for this discrepancy is that all participants in this study were second-class athletes, so it is difficult to determine whether athletic level is a potential confounding factor. Another possible reason is that Mathews et al. (2021) focused on the theme of athletes withdrawing from sports, so the research process involved sport-related psychological factors associated with withdrawal from sports, such as depression, anxiety, and confusion (Giannone et al., 2017), and these factors obviously were adversely associated with perceived wellbeing (Verkooijen et al., 2012). Furthermore, overt adverse outcomes, such as poor competitive performance or sports injuries, may indirectly diminish subjective wellbeing by undermining athletes' sense of self-worth (Brewer et al., 1993).

Additionally, some studies have shown that the years of athletes in school were related to their sense of self-identity (Fuller, 2014). Therefore, it is inferred that years in school may be another factor associated with athletic identity and further correlate with subjective wellbeing (Houle et al., 2010). However, in exploring the relational entity of the impact of ordinary college students' participation in physical exercise on subjective wellbeing, Yao et al. (2025) confirmed that self-identity has a significant positive relationship with subjective wellbeing and simultaneously plays a partial mediating effect in the relationship between physical exercise and subjective wellbeing. This study obviously recruited different research subjects but to a certain extent confirmed the results of this study. The possible reason for analysis is that self-identity achieved during sports was linked to improvements in college students' mental state, especially when they can successfully complete physical exercise tasks, a sense of self-satisfaction will increase accordingly (Sánchez-Miguel et al., 2017). But elite college student-athletes and ordinary college students differ in the purpose of participating in sports, the level of participation, and the sports abilities they possess. Therefore, whether the mechanism of the improvement of subjective wellbeing associated with the identity recognition of these two groups is consistent needs further exploration. Even so, at the macro level, participants achieve self-identity in sports and which was linked to enhanced subjective wellbeing perception, which plays a good theoretical guiding role for reality. In addition, the results of this study further enrich the view of previous studies related to subjective wellbeing of student-athletes from the perspective of social identity (Graupensperger et al., 2020b). The comprehensive development of human beings requires self-identity and social identity, both of which can augment people's psychological resilience toward perceiving happiness (Verkooijen and de Bruijn, 2013). Therefore, the results of this study supplement previous studies from the perspective of student-athletes' self-identity.

Secondly, the results of this study confirmed the mediating roles of perceived team support and athlete self-efficacy in the relational entity of athletic identity and subjective wellbeing, and the mediating effect of perceived team support was higher than that of athlete self-efficacy. Although previous studies explored the impact of athletic identity on happiness or subjective wellbeing from different perspectives, existing studies failed to reveal the mediating mechanisms between the two variables (Mathews et al., 2021; Verkooijen et al., 2012; Yao et al., 2025). Therefore, the sequential mediation model proposed theoretically by the results of this study makes up for the deficiency of existing studies in explaining the mechanism of action between the two variables.

Specifically, this study found two important mediating variables with a sequential relationship, namely perceived team support and athlete self-efficacy, which positively and sequentially connect student-athletes‘ athletic identity and subjective wellbeing. This research result is supported to a certain extent by previous studies. Cho et al. (2020) found in a survey of college student-athletes that support from teammates can significantly was associated with the expression of subjective wellbeing. At the same time, positive emotions associated with by teammate support, such as happiness, pride, enthusiasm, etc., can also significantly relate to college student-athletes' subjective wellbeing. Different from that study which treated perceived team support as a predictor variable, this study treats this variable as a mediating variable to test the mediating mechanism of the impact of athlete self-identity on subjective wellbeing.

Therefore, to a certain extent, the theoretical model of this study is a further exploration of it. In addition, a study focused on the moderating effect of perceived social support on subjective wellbeing from the perspective of moderation, arguing that good perceived social support can effectively moderate the moderating effect of self-esteem on neuroticism on subjective wellbeing in young professional athletes (Solmaz, 2025). Social support usually includes emotional support, esteem support, informational support, and tangible support (Rees and Hardy, 2000). In the context of sports, although family and friends also provide support, team members, such as teammates and coaches, are more important sources of support (Cho et al., 2020). Therefore, it can be inferred that perceiving team support has a positive impact on elite college student-athletes' expression of subjective wellbeing in different research contexts. Compared with the moderating effect of perceived team support (Solmaz, 2025), this study explored the role of this variable theoretically and empirically from the perspective of mediation, thus demonstrating certain academic value.

In recent years, studies have focused on the influence of self-efficacy on the expression of subjective wellbeing in sports (Guo et al., 2024; Wang et al., 2022; Yiming et al., 2024). However, unlike recent studies, this study first confirmed that self-efficacy plays a mediating effect in the relationship between athletic identity and subjective wellbeing among elite college student-athletes. This influencing mechanism may find support from previous studies. For example, Wang et al. (2022) found in a survey of ordinary college students that self-efficacy plays a mediating effect in the relationship between physical exercise and subjective wellbeing. The possible reason is that participation in physical exercise, on the one hand, can increase interactions among students to link to social interaction (Smith, 2003), and on the other hand, exercise itself can bring a certain sense of pleasure to participants (Wang et al., 2022). These are exactly the mental health factors that influence the generation of self-efficacy (Wang et al., 2022), and at the same time, these factors themselves can also link to the positive expression of subjective wellbeing (Gangying and Qian, 2016). Additionally, Guo et al. (2024) also found through an empirical study of ordinary college students' participation in sports that self-efficacy is not only influenced by participation in sports but also indirectly associate with students' evaluation of subjective wellbeing. The study found that physical exercise can positively relate to college students' willpower, optimism, and sense of competence related to self-efficacy, thereby promoting students' confidence in their own abilities to fortify subjective wellbeing. It can be seen that the influence of self-efficacy on the evaluation of subjective wellbeing is manifested as their relatively satisfied evaluation of their own living conditions with the positive improvement of the respondents' internal psychological state. In this study, elite college student-athletes can clearly recognize and acknowledge their self-roles and positioning in training or competitions, so they can excellently complete the tasks of corresponding roles in training and competitions. This experience linked to their experience of a sense of competence, thereby enhancing self-efficacy and the sense of wellbeing brought by successfully completing competition and training tasks. However, whether this mechanism of action is generalizable across different cultures and different sports, we suggest conducting further empirical studies.

Additionally, an important finding of this study is that the mediating effect of perceived team support is higher than that of athlete self-efficacy. Unfortunately, in existing studies, we failed to find literature to explain this phenomenon. Therefore, we attempted to use two concepts to explain the existing difference. According to self-efficacy theory (Bandura, 1977), self-efficacy refers to a person's belief in their ability to accomplish something in certain aspects, so as to be able to control events and influence their own life. It can be seen that self-efficacy is the “belief in one's capabilities to organize and execute the courses of action required to produce given attainments.” While perceived team support is a microcosm of perceived social support, it is generally believed that perceived social support is comprehensive psychological and material support from an individual's social network, helping individuals cope with stress and various challenges (Cohen, 2004). Then, for elite college student-athletes, perceived team support usually refers to support from relevant personnel within the team, such as coaches, teammates (Solmaz, 2025). It can be seen that perceived team support is people seeking external help. Studies suggest that external social support is a more effective factor in buffering stressors (Sullivan et al., 2020). Typically, the feeling of receiving support from others helps individuals cultivate a positive emotional state and thereby amplifies their psychological response to stress (Cohen, 2004). Through observation, we found that young athletes usually seek help from peers when completing team tasks. Therefore, we infer that this is the reason why the mediating effect of perceived team support is higher than that of athlete self-efficacy. Furthermore, drawing upon Conservation of Resources theory (Hobfoll, 1989; Hobfoll et al., 2018), we posit that external social resources, specifically the perceived team support examined in this study, serve as critical buffers against stress by providing both tangible and emotional sustenance. For emerging adult athletes, the team functions as a primary developmental context in which collective efficacy and social identity often supersede individual agency in regulating stress. Consequently, the more potent mediating effect of perceived team support aligns with its capacity to mobilize communal resources, thereby offering a more robust protective mechanism than solitary self-belief.

A notable finding from our mediation analysis is that the indirect effect of athletic identity on subjective wellbeing via perceived team support alone was significantly stronger than the sequential mediation path through both team support and self-efficacy. We speculate that this is largely attributable to the nature of the sample and the cultural context. As elite collegiate athletes in China, these individuals operate within a highly structured, collectivist sporting system where the team functions as the primary developmental context. By referring to conservation of resources theory (Hobfoll, 1989; Hobfoll et al., 2018), perceived team support represents a critical external resource that provides immediate, tangible, and emotional sustenance. For these athletes, the sense of belonging and validation from coaches and teammates may serve as a more potent buffer against competitive stress than internal self-beliefs. Furthermore, the transition from team support to self-efficacy might involve complex intrapsychic processes that dilute the total effect size. It appears that while team support is vital, its translation into a stable sense of personal competence (self-efficacy) may be less impactful than the direct psychological comfort derived from simply feeling supported within the team environment (Solmaz, 2025).

Finally, the sequential mediation model proposed in this study reveals the possibility of different paths in the relationship between elite college student-athletes' athletic identity and subjective wellbeing perception, providing new insights for relevant literature. Compared with previous studies that focused only on a single variable, such as perceived team support (Cho et al., 2020) and self-efficacy (Guo et al., 2024; Wang et al., 2022; Yiming et al., 2024), this study first explores these two variables that influence the expression of subjective wellbeing as sequential mediators. The results provide theoretical support for the management of team sports programs and also have practical implications, Such as organizing team-building workshops, implementing cognitive restructuring techniques to boost support and self-efficacy.

## 6 Limitations

In the design and implementation of this study, there are several limitations. First, the student-athletes participating in the survey of this study were all national second-class athletes, which prevented us from determining whether the difference in athletic levels (Mathews et al., 2021) would affect the relational entity of athletic identity on subjective wellbeing. It is suggested that future studies include student-athletes of diverse levels for verification. Second, although this study took team sports as the research entry point, the survey showed that the sports mainly included football, basketball, volleyball, and a small part of hockey. Therefore, whether the corresponding research conclusions are applicable to other sports requires further empirical evidence. Third, given the large gender ratio of participants in the study, we did not examine the differential effect of gender in the influence mechanism of athletic identity and subjective wellbeing. Therefore, we do not know whether gender differences have different manifestations in the influence mechanism. It is suggested that future studies include the factor of gender. Fourth, although this study recognized the differences in the mediation mechanism, it is suggested that future studies deeply explore the potential deep-seated reasons from the perspective of qualitative methods. Fifth, as self-reported social support and wellbeing may inflate correlations. Future research should adopt multi-source designs, collecting team support from coaches and wellbeing via objective biomarkers to mitigate common method variance. The exceptionally high ICC(1) values observed in this study suggest a potential limitation regarding the generalizability of our findings. Specifically, the restricted within-team variance implies that individual-level differences among our participants were inherently limited. This constraint is likely attributable to the extreme homogeneity characteristic of our elite sample. Consequently, while team-level aggregation proved analytically appropriate for the constructs examined here, the findings should be interpreted with caution when considering broader or more heterogeneous athletic populations. Future studies involving more diverse competitive levels are needed to establish the generalizability of these mechanisms beyond elite samples. Last but not least, a key limitation of this study is its cross-sectional design, which collects data at a single time point. As such, we cannot establish temporal precedence or rule out reverse causality. All reported associations should be interpreted as correlational rather than causal. Future research employing longitudinal designs or experimental interventions is needed to validate directional relationships.

## 7 Conclusion

This study examines the chain-mediated relationship between athletic identity and subjective wellbeing among elite collegiate athletes within a team sports context. Our findings confirm a significant positive association between these constructs, with perceived team support and self-efficacy acting as key sequential mediators. By mapping out these interconnected pathways, we address a gap in the current literature regarding how athletic identity translates into wellbeing. Theoretically, these results illuminate the mechanisms linking identity to psychological outcomes, offering a foundation for future research on athlete mental health. Practically, the findings suggest that stakeholders, including program directors, coaches, and athletes themselves, can better safeguard athlete welfare by fostering supportive team environments and bolstering self-efficacy beliefs.

## Data Availability

The original contributions presented in the study are included in the article/supplementary material, further inquiries can be directed to the corresponding author.
